# Quantitative double echo steady state T2 mapping of upper extremity peripheral nerves and muscles

**DOI:** 10.3389/fneur.2024.1359033

**Published:** 2024-02-15

**Authors:** Gracyn J. Campbell, Darryl B. Sneag, Sophie C. Queler, Yenpo Lin, Qian Li, Ek T. Tan

**Affiliations:** ^1^Department of Radiology and Imaging, Hospital for Special Surgery, New York, NY, United States; ^2^College of Medicine, Downstate Health Sciences University, Brooklyn, NY, United States; ^3^Department of Medical Imaging and Intervention, Chang Gung Memorial Hospital, Taoyuan City, Taiwan; ^4^Biostatistics Core, Hospital for Special Surgery, New York, NY, United States

**Keywords:** quantitative MRI, peripheral neuropathy, T2 mapping, deep learning reconstruction, magnetic resonance neurography

## Abstract

**Introduction:**

T2 mapping can characterize peripheral neuropathy and muscle denervation due to axonal damage. Three-dimensional double echo steady-state (DESS) can simultaneously provide 3D qualitative information and T2 maps with equivalent spatial resolution. However, insufficient signal-to-noise ratio may bias DESS-T2 values. Deep learning reconstruction (DLR) techniques can reduce noise, and hence may improve quantitation of high-resolution DESS-T2. This study aims to (i) evaluate the effect of DLR methods on DESS-T2 values, and (ii) to evaluate the feasibility of using DESS-T2 maps to differentiate abnormal from normal nerves and muscles in the upper extremities, with abnormality as determined by electromyography.

**Methods and results:**

Analysis of images from 25 subjects found that DLR decreased DESS-T2 values in abnormal muscles (DLR = 37.71 ± 9.11 msec, standard reconstruction = 38.56 ± 9.44 msec, *p* = 0.005) and normal muscles (DLR: 27.18 ± 6.34 msec, standard reconstruction: 27.58 ± 6.34 msec, *p* < 0.001) consistent with a noise reduction bias. Mean DESS-T2, both with and without DLR, was higher in abnormal nerves (abnormal = 75.99 ± 38.21 msec, normal = 35.10 ± 9.78 msec, *p* < 0.001) and muscles (abnormal = 37.71 ± 9.11 msec, normal = 27.18 ± 6.34 msec, *p* < 0.001). A higher DESS-T2 in muscle was associated with electromyography motor unit recruitment (*p* < 0.001).

**Discussion:**

These results suggest that quantitative DESS-T2 is improved by DLR and can differentiate the nerves and muscles involved in peripheral neuropathies from those uninvolved.

## Introduction

Peripheral neuropathies are caused by nerve compression, inflammation, or trauma and may lead to muscle denervation if axonal injury ensues (1, 2). Magnetic resonance neurography (MRN) techniques employ T2-weighted, fat-suppressed sequences with flow suppression to depict nerve morphology and to highlight pathologically increased signal of both nerves and muscles (3). Conventional T2 mapping provides quantitative information with potentially less bias than qualitative evaluation of T2-weighted signal intensity (4). T2 mapping can quantify neuropathy-related changes in nerve (5, 6) and has demonstrated correlation with electrodiagnostic results in muscles including those of the upper extremity (4, 7).

While T2 values may be obtained using a number of different methods, including fast spin-echo (FSE)/turbo spin-echo (TSE) at multiple echo times (5) and T2-prepared TSE (8), the most commonly-used method for T2-mapping is the conventional multi-echo spin-echo (MESE) method. While T2 mapping using MESE has been readily applied in muscle (7, 9), it is limited in nerve imaging because (i) as a 2D acquisition, MESE typically has poor slice-resolution (3–6 mm) and may not be readily reformatted in different planes for nerve evaluation, and (ii) MESE has poor scan efficiency, requiring multiple echoes (8–16) and often long scan times (>8 min) for acquiring high in-plane resolution (0.3 mm) (10). Nevertheless, promising findings of correlation between nerve MESE T2 and electrodiagnostic results have been observed (4, 11), which motivate improving the spatial resolution of T2 mapping in peripheral nerve assessment.

Double echo steady-state (DESS) can provide high-resolution, T2-weighted images for morphologic nerve assessment (12, 13). DESS can also simultaneously generate T2 maps at identical spatial resolutions (12, 14), eliminating the need for image registration (10) and reducing the overall exam time if T2 maps are also desired. However, as high spatial resolutions are typically acquired in MRN (~0.3 mm in-plane, ~1.6 mm through-plane), and signal-to-noise ratio (SNR) decreases with resolution, insufficient SNR may still potentially bias DESS-T2 values.

Deep learning reconstruction (DLR) techniques have demonstrated the ability to improve image quality (15–18). In 2D imaging, DLR reduces noise, blurring, and Gibbs ringing (19–22), which in MRN, improves peripheral nerve conspicuity and qualitative assessment (23). 3D-DLR has been applied in spine MRI and brachial plexus MRN, demonstrating improved image quality compared to standard reconstruction (24–28), but has not been demonstrated in DESS-T2 mapping.

This study aims to (i) evaluate the effect of 3D-DLR on DESS-T2 values, and (ii) to evaluate the feasibility of using DESS-T2 maps to differentiate pathologic from normal nerves and muscles in the upper extremities. We hypothesized that DLR would reduce mean T2 due to a reduction in Rician noise bias (29). We also hypothesized that mean T2 would be higher in abnormal nerves and muscles, and that DESS-T2 would be associated with electromyography (EMG) results.

## Materials and methods

### Study subjects and power analysis

This study was approved by the institutional review board and written informed consent waived due to its retrospective nature. Electronic medical records were searched for patients who underwent standard, clinical MRN with DESS to evaluate suspected neuropathy localized to the elbow or forearm region, between July 2022 and April 2023.

**Figure 1:**
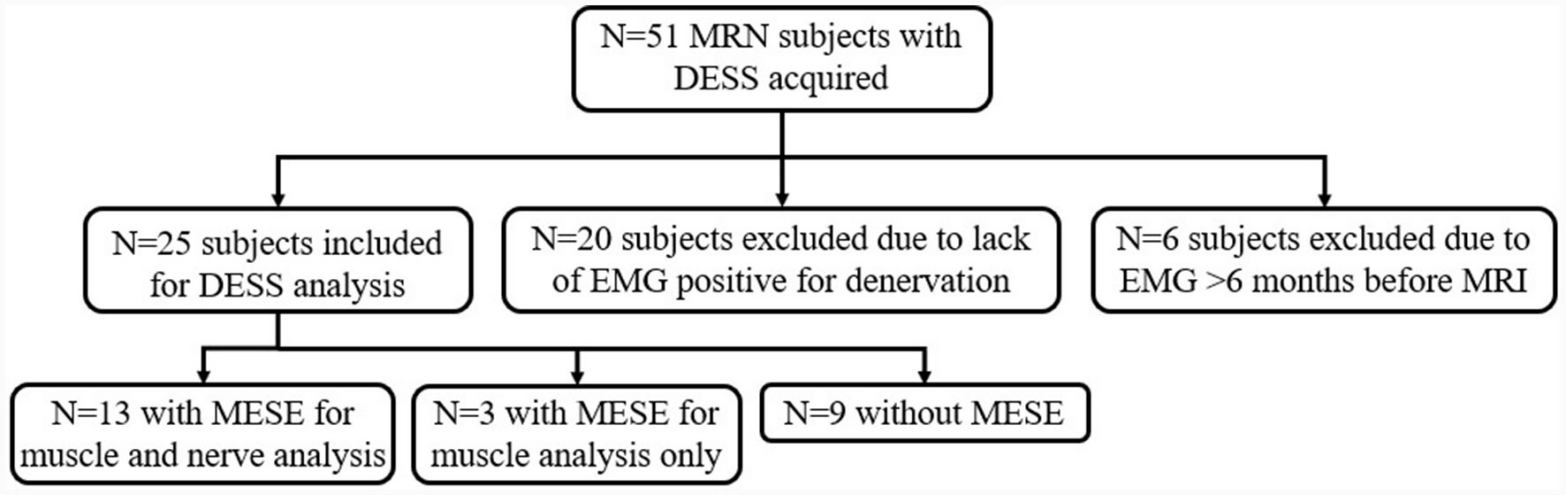
Flow diagram of subject inclusion, reasons for exclusion, and subjects analyzed.

Patients met inclusion criteria if they underwent an EMG that showed abnormal results in at least one of the median, radial, or ulnar nerve distributions (Figure 1). Abnormality was defined as the presence of denervation potentials or abnormal motor unit recruitment (MUR) on EMG. EMGs performed outside of a 6-month interval from MRN were excluded, similar to a prior study (7).

Based on a previous study involving T2 mapping in forearm nerve compression (5), it was calculated that the current study would require a sample size of 16 to achieve a power of 80% and a level of significance of 5% (two-sided) for detecting a mean T2 difference of 5.5 msec between pairs, assuming the standard deviation of the differences was 6.9 msec.

### Clinical EMG

EMGs were assessed for (i) MUR, in increasing severity from “full” to “reduced,” “discrete,” and “none,” and (ii) denervation potentials in the form of fibrillation potentials (FPs) or positive sharp waves (PSWs) (30). As both FPs and PSWs are measures of abnormal spontaneous activity with increasing severity from “0” (no denervation) to “1+”, “2+”, “3+” and “4+”, for each muscle only the more severe of FPs and PSWs was used for analysis (31).

### Image acquisition

All MRN was performed at 3 Tesla (Premier, GE Healthcare, Waukesha, WI). The imaging protocol (Supplementary Table 1) included an axial, 3D-DESS sequence [also known as Multi-Echo iN Steady-State Acquisition (MENSA), resolution = 0.3 × 0.3 × 1.6 mm, scan time = 4–6 min]. If conventional T2 mapping results were also available, acquired as part of other muscle edema studies (2D axial MESE, 0.6 × 0.7 × 4.0 mm, 5 min), these were included for comparison against DESS-T2 maps.

### Image reconstruction

3D-DESS images were reconstructed with standard image reconstruction and with 3D-DLR as part of an offline pipeline (25). For both reconstructions, the two DESS echoes were separated to generate DESS-T2 maps. A vendor-provided prototype DLR software (Air™ Recon DL, GE Healthcare, Waukesha, WI, USA) was utilized to reduce noise and improve sharpness by processing 3D data for de-ringing, denoising, and interpolation in all three directions (21, 25). The algorithm uses a convolutional neural network of 4.4 million parameters in approximately 10,000 kernels trained with a supervised learning approach using pairs of conventional and ‘near perfect' (high resolution, low noise, and minimal ringing) images. The software, which operates on raw, complex-valued datasets, allows for the selection of the extent of DLR—either “low” (25%,), “medium” (50%) or “high” (75%); DLR “high” was utilized and implemented on a separate workstation (Ubuntu 20.04, Intel Xeon W-2265 CPU, Nvidia RTX A5000 GPU, reconstruction time: 2 min) (21, 25).

### T2 mapping

**Figure 2:**
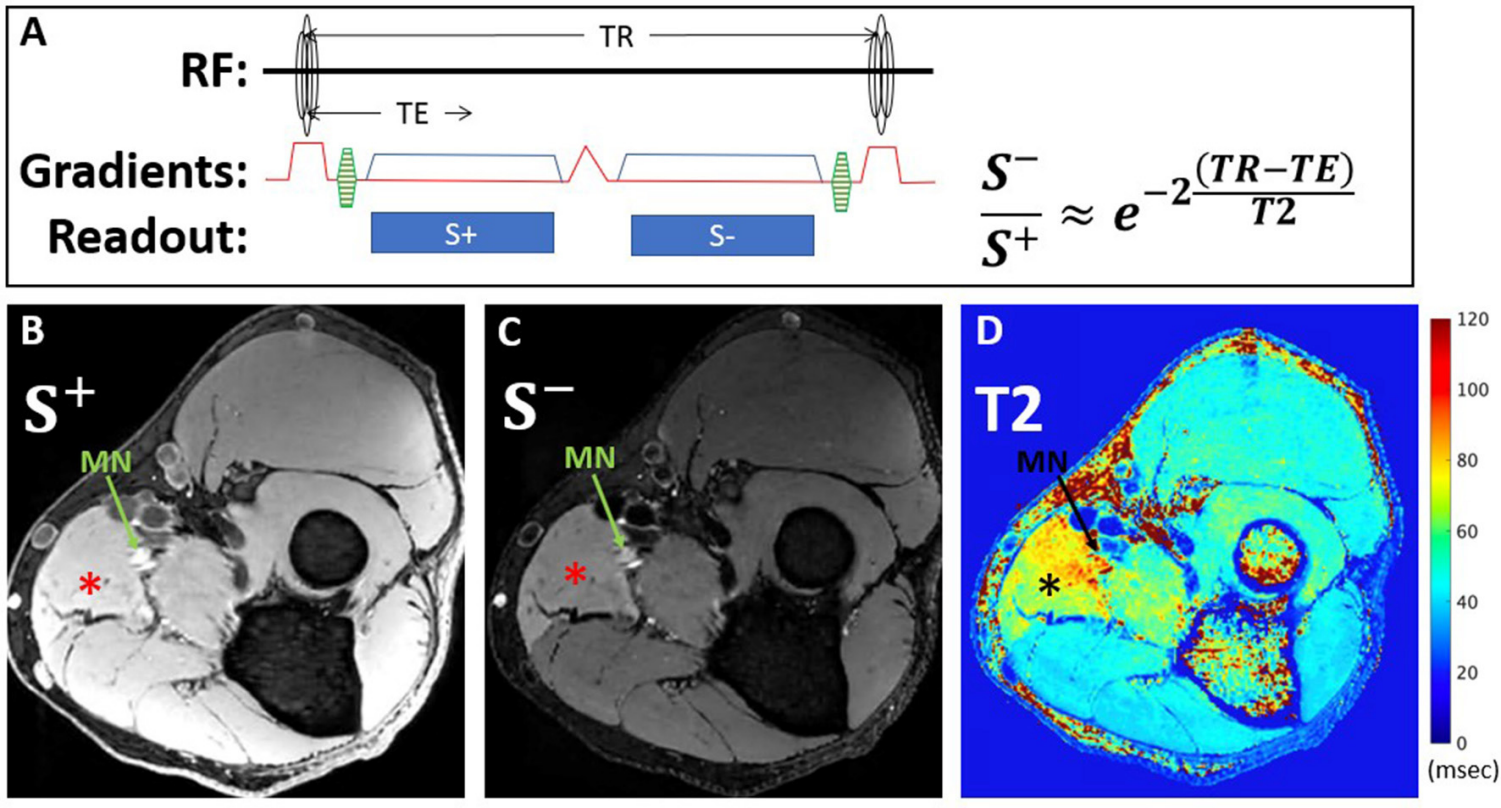
DESS pulse sequence diagram and equation used for T2 calculation **(A)**. The first echo, S+, utilizes free induction decay signal and has mixed T1 and T2 contrast, while the second echo, S-, is more heavily T2-weighted, due to contributions from previous excitations. Representative axial images from a 39-year-old man with Parsonage-Turner Syndrome of S+ **(B)** and S- **(C)** echoes acquired within the proximal forearm reveal diffuse hyperintensity, compatible with active denervation, of the pronator teres muscle (^*^), best seen on the S- image, and also of the median nerve (MN, arrow). Corresponding DESS-T2 map **(D)** demonstrates increased T2 in the pronator teres and median nerve.

DESS is a steady-state free precession sequence with its two echoes separated by a spoiler gradient (Figure 2). The first echo, *S*^+^, utilizes free induction decay signal and has mixed T1 and T2 contrast. The second echo, *S*^−^, is more heavily T2-weighted than the first echo, due to contributions from previous excitations. Equations describing the signal of both echoes based on the T1, T2, TR, and TE (32, 33) show that DESS-T2 is relatively invariant to T1 (14), and from these the T2 may be approximated using Equation 1:


(1) $$\begin{array}{l} \frac{S^{-}}{S^{+}}=e^{-2\frac{\left(TR-TE\right)}{T2}} \end{array}$$

As these approximated T2 values can slightly underestimate the actual T2 values from DESS equations (33), these approximated T2 values were then mapped to the actual T2 values, using a dictionary of T2 values from 3 ms to 300 ms in steps of 0.1 ms, assuming nerve T1 = 1,600 ms, and muscle T1 = 1,400 ms. The DESS-T2 maps were obtained separately for standard reconstruction and DLR images using in-house code (https://github.com/hssmri/mensa.git, MATLAB, MathWorks Inc., Natick, MA).

Conventional T2-mapping was also processed using in-house code developed on MATLAB for muscle analysis, using single-exponential fitting with B_1_+ correction (34).

### Quantitative image analysis

**Figure 3:**
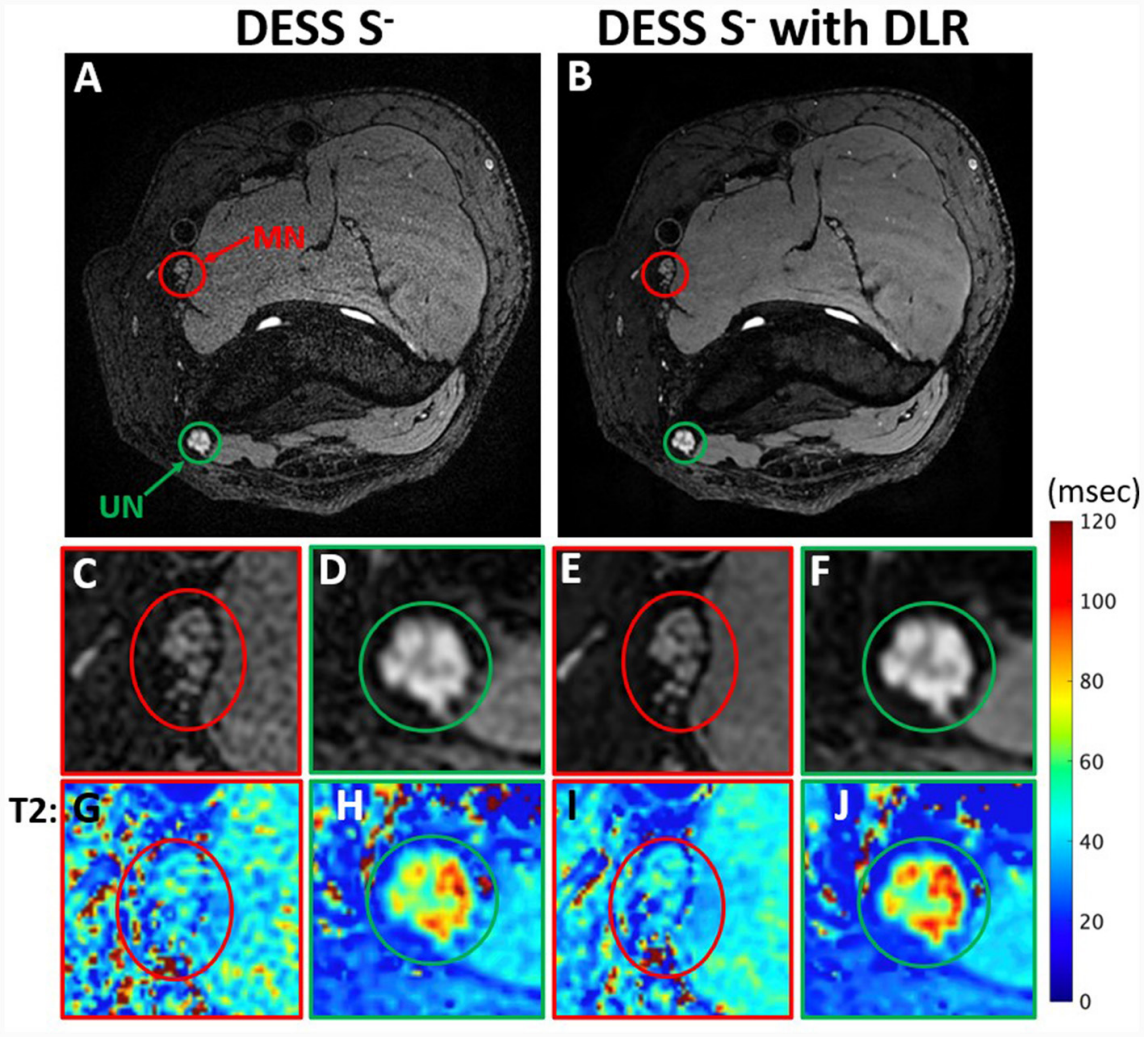
Comparison of DESS S^−^ images with standard reconstruction **(A)** and with DLR **(B)** from a 44-year-old man with ulnar neuropathy. Insets show reduced noise and superior conspicuity with DLR of fascicular bundles in the normal median nerve (MN) **(C, E)** and abnormal ulnar nerve (UN) **(D, F)**. The effect of DLR is also seen in T2 map insets **(G–J)** corresponding to **(C–F)**.

**Figure 4:**
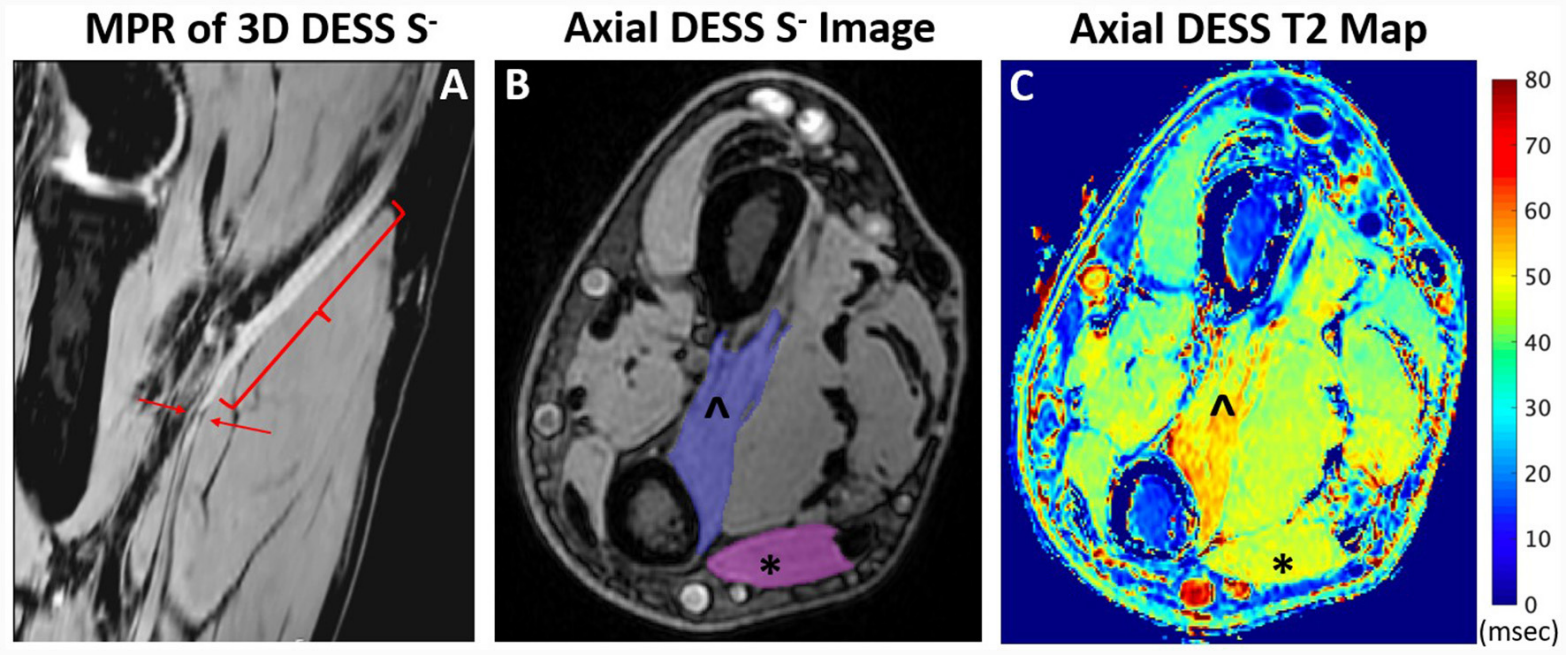
Oblique coronal multiplanar reformat (MPR) of 3D DESS acquired at the elbow joint **(A)** shows enlargement and hyperintensity (bracket) and a focal constriction (arrows) of the median nerve in a 48-year-old man, compatible with Parsonage-Turner syndrome. Axial DESS S- image **(B)** through the distal forearm shows muscle ROI selection of the pronator quadratus (PQ) (∧) and flexor carpi ulnaris (FCU) (^*^). T2 map **(C)** through the distal forearm shows increased T2 in the denervated PQ compared to the normal FCU.

For each subject, one abnormal nerve and muscle, and one normal nerve and muscle were identified, based on EMG evaluation. Regions of interest (ROIs) were manually segmented on DESS images and checked for alignment with MESE images (where available) using ITK-Snap (35) by two raters—a research assistant (GC/Rater 1 with 1 year of image segmentation experience) and a second-year medical student (SQ/Rater 2 with 4 years' experience). The abnormal nerve ROI was selected on three non-consecutive *S*^−^ axial image slices corresponding to the area of nerve abnormality. The normal nerve ROI was then selected on the same slices (Figure 3). Abnormal and normal muscles were similarly segmented on three non-consecutive image slices, with denervated muscles typically distal to the involved nerve (Figure 4). Image ROIs were checked by a radiologist (YL, with seven years' experience) to confirm that placement of ROIs agreed with reports of either abnormality or normality on EMG. The mean T2 and its standard deviation normalized to the mean were obtained for each nerve or muscle ROI.

### Statistical analysis

Pearson's correlation (r) was used to evaluate inter-rater agreement for all measurements. One-way repeated measures ANOVA was used to compare the mean DESS-T2 and T2 standard deviation of four groups of a within-subjects variable (normal nerve/muscle with standard reconstruction, abnormal nerve/muscle with standard reconstruction, normal nerve/muscle with DLR, abnormal nerve/muscle with DLR). Multiple pairwise paired *t*-tests were next performed to examine the differences in mean T2 and T2 standard deviation between different permutations of pairs between the four groups. The *p*-values were adjusted using the Bonferroni multiple testing correction method. Kendell's Rank Correlation and generalized linear models were used to evaluate associations between mean T2 and either FPs/PSWs or MUR. All statistical analyses were performed using R (version 4.0.3). A *p* < 0.05 was considered statistically significant.

### Magic angle dependency

The magic angle behavior of nerve DESS-T2 with DLR was also evaluated. The nerve orientation with respect to B_0_ was determined by Rater 1 using Volume Viewer (GE Healthcare, Waukesha, WI, USA), which allowed vector lines to be drawn tangent to the nerve, from which the solid angle from B_0_ could be obtained via the cosine rule (Supplementary Figure 1). Linear regressions between this calculated angle and DESS-T2 were performed. This was conducted separately for abnormal and normal nerves.

## Results

Of the 51 patients initially identified who had undergone MRN with DESS, 20 were excluded because they did not have EMG results indicating denervation, and six more were excluded as the EMG was performed beyond the requisite 6-month interval from MRI (Figure 1). Demographic characteristics for all 25 patients who met inclusion criteria (52 ± 15 years, 15 men) are listed in Table 1. Among these, 16 were diagnosed with Parsonage-Turner syndrome, three with non-specific radial neuropathy, three with cubital tunnel syndrome (ulnar nerve), one with inflammatory neuropathy involving the radial and median nerves, one with pronator teres syndrome (median nerve), and one with idiopathic wrist drop (radial nerve). EMGs were performed by one of nine providers; a single provider with >30 years' electrodiagnostic experience performed 13 evaluations. In 16 of the 25 subjects, an additional MESE sequence was acquired as part of a different prospective study in Parsonage-Turner syndrome, where the MESE was acquired for muscle analysis rather than for the nerves. Hence, only 13 of 16 MESE data sets could be analyzed for nerve involvement, due to the limited slice coverage obtained.

**Table 1:** Patient demographics.

**Demographics**		***N***
Mean age, years (range)		52 (22–80)
Sex (%)	Female	10 (40)
	Male	15 (60)
Upper extremity (%)	Left	8 (32)
	Right	17 (68)
Days from EMG to MRI [mean (SD)]		18.2 (25.78)
Abnormal nerve (%)	Median nerve	15 (60)
	Radial nerve	7 (28)
	Ulnar nerve	3 (12)
Abnormal muscle (%)	Pronator teres	6 (24)
	Extensor digitorum	6 (24)
	Flexor digitorum profundus	5 (20)
	Flexor pollicis longus	3 (12)
	Pronator quadratus	2 (8)
	Flexor digitorum superficialis	2 (8)
	Flexor carpi ulnaris	1 (4)

EMG, Electromyography; SD, standard deviation.

### Inter-rater agreement for quantitative T2

The Pearson *r* correlation coefficients of mean T2 and T2 standard deviation measurements between the two raters ranged from 0.864 to 0.998 (Table 2). Due to this high degree of correlation, the mean value of both readers was used for subsequent comparisons.

**Table 2:** Inter-rater agreement of quantitative measurements.

**Variable**	**Correlation coefficient, *r***	***p*-value**
**Mean T2**		
Abnormal Nerve DESS-T2 with Standard Reconstruction	0.991	**< 0.001**
Normal Nerve DESS-T2 with Standard Reconstruction	0.948	**< 0.001**
Abnormal Nerve DESS-T2 with DLR	0.993	**< 0.001**
Normal Nerve DESS-T2 with DLR	0.966	**< 0.001**
Abnormal Muscle DESS-T2 with Standard Reconstruction	0.992	**< 0.001**
Normal Muscle DESS-T2 with Standard Reconstruction	0.998	**< 0.001**
Abnormal Muscle DESS-T2 with DLR	0.989	**< 0.001**
Normal Muscle DESS-T2 with DLR	0.998	**< 0.001**
**Normalized T2 Standard Deviation**		
Abnormal Nerve DESS-T2 with Standard Reconstruction	0.979	**< 0.001**
Normal Nerve DESS-T2 with Standard Reconstruction	0.864	**< 0.001**
Abnormal Nerve DESS-T2 with DLR	0.977	**< 0.001**
Normal Nerve DESS-T2 with DLR	0.945	**< 0.001**
Abnormal Muscle DESS-T2 with Standard Reconstruction	0.897	**< 0.001**
Normal Muscle DESS-T2 with Standard Reconstruction	0.913	**< 0.001**
Abnormal Muscle DESS-T2 with DLR	0.869	**< 0.001**
Normal Muscle DESS-T2 with DLR	0.916	**< 0.001**

DLR, Deep learning reconstruction; DESS, double echo steady state. Statistically significant results (*p* < 0.05) are in bold font.

### DLR vs. standard reconstruction

**Figure 5:**
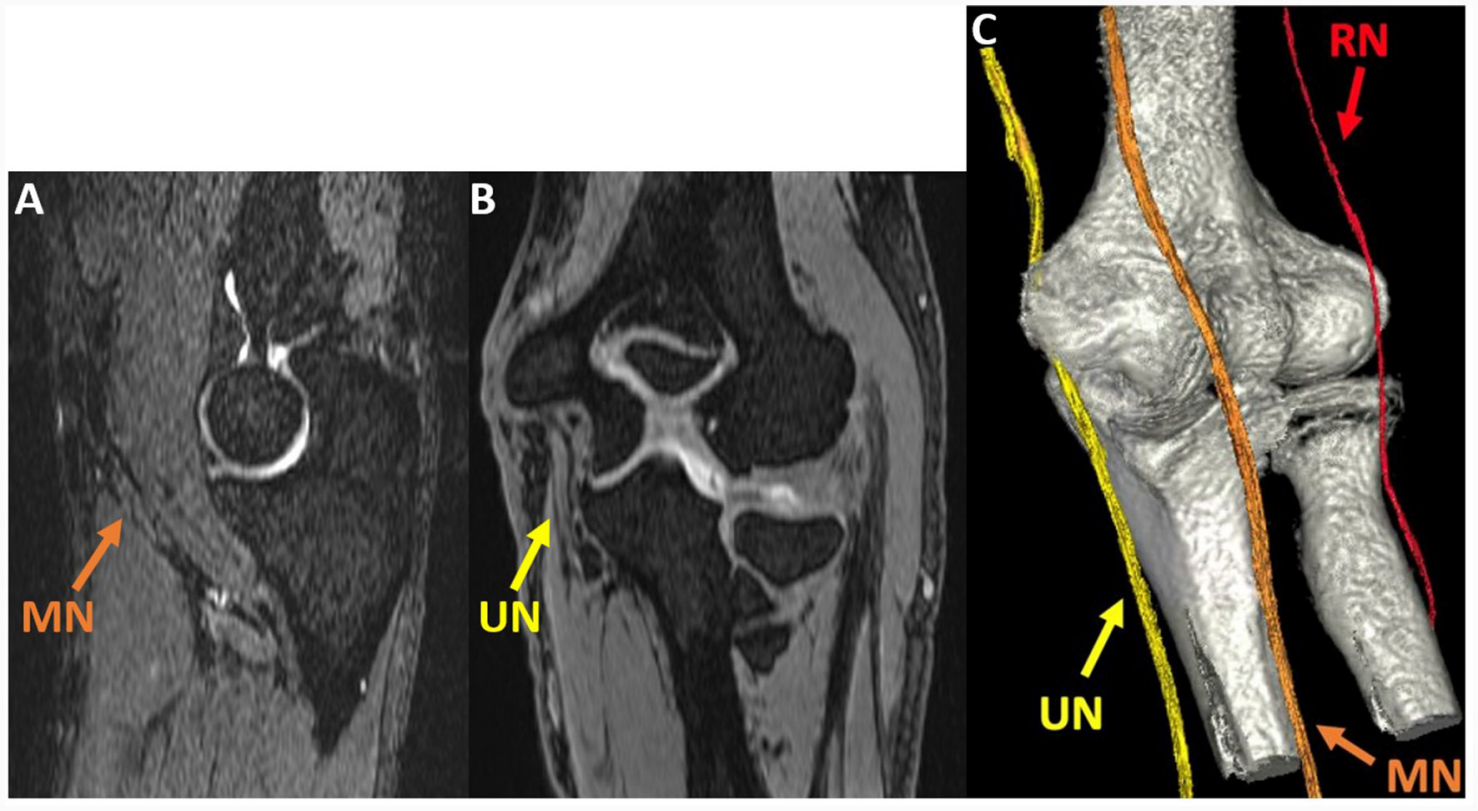
Oblique sagittal **(A)** and coronal **(B)** multiplanar reformat (MPR) of the 3D DESS S- echo, acquired at the elbow joint in a 51-year-old man with cubital tunnel syndrome, show the median (MN) and ulnar nerves (UN). A 3D rendering **(C)** generated using 3D DESS for nerves and a zero time-to-echo sequence for bones allows for visualization of the MN, UN, and radial nerve (RN).

Abnormal nerves analyzed were the median (16), radial (5), and ulnar nerves (4) (Figure 5). Table 3 shows that the mean T2 was significantly lower with DLR than with standard reconstruction for abnormal muscles (DLR: 37.71 ± 9.11 msec, standard reconstruction: 38.56 ± 9.44 msec, *p* = 0.005) and normal muscles (DLR: 27.18 ± 6.34 msec, standard reconstruction: 27.58 ± 6.34 msec, *p* < 0.001). Differences in abnormal and normal nerves between standard reconstruction and DLR were not significant (*p* = 1 for both). The T2 mean-normalized standard deviation (no units) was not significantly different with DLR compared to standard reconstruction for muscles or nerves (Table 4).

**Table 3:** Comparison of mean T2 between standard reconstruction and DLR and between abnormal nerves/muscles and normal nerves/muscles.

**Variable**	***N***	**Mean (ms)**	**SD**	**Multiple comparisons (paired** ***t*****-test with adjusted** ***p*****-values)**		
				**Group 1**	**Group 2**	***p*** **-value**
**Nerve**						
Abnormal (DLR)	25	75.99	38.21	Abnormal (DLR)	Abnormal (standard recon.)	1
Abnormal (standard recon.)	25	74.32	37.66	Abnormal (DLR)	Normal (DLR)	**< 0.001**
Normal (DLR)	25	35.10	9.78	Abnormal (DLR)	Normal (standard recon.)	**< 0.001**
Normal (standard recon.)	25	34.49	7.93	Abnormal (standard recon.)	Normal (DLR)	**< 0.001**
**ANOVA** ***p*****-value: 0.008**				Abnormal (standard recon.)	Normal (standard recon.)	**<** **0.001**
				Normal (DLR)	Normal (standard recon.)	1
**Muscle**						
Abnormal (DLR)	25	37.71	9.11	Abnormal (DLR)	Abnormal (standard recon.)	**0.005**
Abnormal (standard recon.)	25	38.56	9.44	Abnormal (DLR)	Normal (DLR)	**< 0.001**
Normal (DLR)	25	27.18	6.34	Abnormal (DLR)	Normal (standard recon.)	**< 0.001**
Normal (standard recon.)	25	27.58	6.34	Abnormal (standard recon.)	Normal (DLR)	**< 0.001**
**ANOVA** ***p*****-value:** ** < 0.001**				Abnormal (standard recon.)	Normal (standard recon.)	**<** **0.001**
				Normal (DLR)	Normal (standard recon.)	**< 0.001**

DLR, Deep learning reconstruction; recon., reconstruction. Statistically significant results (p < 0.05) are in bold font.

**Table 4:** Comparison of mean normalized T2 standard deviation between standard reconstruction and DLR and between abnormal nerves/muscles and normal nerves/muscles.

**Variable**	***N***	**Mean (NU)**	**SD**	**Multiple comparisons (paired** ***t*****-test with adjusted** ***p*****-values)**		
				**Group 1**	**Group 2**	***p*** **-value**
**Nerve**						
Abnormal (DLR)	25	0.404	0.209	Abnormal (DLR)	Abnormal (standard recon.)	1
Abnormal (standard recon.)	25	0.401	0.214	Abnormal (DLR)	Normal (DLR)	0.058
Normal (DLR)	25	0.272	0.181	Abnormal (DLR)	Normal (standard recon.)	**0.034**
Normal (standard recon.)	25	0.266	0.156	Abnormal (standard recon.)	Normal (DLR)	0.124
**ANOVA** ***p*****-value:** ** < 0.001**				Abnormal (standard recon.)	Normal (standard recon.)	0.058
				Normal (DLR)	Normal (standard recon.)	1
**Muscle**						
Abnormal (DLR)	25	0.372	0.166	Abnormal (DLR)	Abnormal (standard recon.)	0.732
Abnormal (standard recon.)	25	0.391	0.175	Abnormal (DLR)	Normal (DLR)	**0.002**
Normal (DLR)	25	0.24	0.168	Abnormal (DLR)	Normal (standard recon.)	**0.016**
Normal (standard recon.)	25	0.257	0.179	Abnormal (standard recon.)	Normal (DLR)	**< 0.001**
**ANOVA** ***p*****-value:** ** < 0.001**				Abnormal (standard recon.)	Normal (standard recon.)	**0.003**
				Normal (DLR)	Normal (standard recon.)	1

DLR, Deep learning reconstruction; SD, standard deviation; NU, no units; recon., reconstruction. Statistically significant results (p < 0.05) are in bold font.

### Normal vs. abnormal

Table 3 shows that mean DESS-T2 was higher for abnormal nerves than normal nerves with standard reconstruction (abnormal: 74.32 ± 37.66 msec, normal: 34.49 ± 7.93 msec, *p* < 0.001) and with DLR (abnormal: 75.99 ± 38.21 msec, normal: 35.10 ± 9.78 msec, *p* < 0.001). Mean DESS-T2 was also higher in abnormal muscles than normal muscles with both standard reconstruction (abnormal: 38.56 ± 9.44 msec, normal: 27.58 ± 6.34 msec, *p* < 0.001) and DLR (abnormal: 37.71 ± 9.11 msec, normal: 27.18 ± 6.34 msec, *p* < 0.001).

### MESE vs. DESS

Linear regression found significant associations between MESE and DESS (both with DLR and with standard reconstruction) for abnormal muscles (standard reconstruction: slope = 1.044, *r* = 0.801; DLR: slope = 1.007, *r* = 0.794) and normal nerves (standard reconstruction: slope = 0.426, *r* = 0.471; DLR: slope = 0.405, *r* = 0.436). Associations for normal muscles (standard reconstruction: *r* = 0.260, DLR: *r* = 0.276) and abnormal nerves (standard reconstruction: *r* = 0.067, DLR: *r* = 0.059) were not significant.

### Associations with EMG

**Figure 6:**
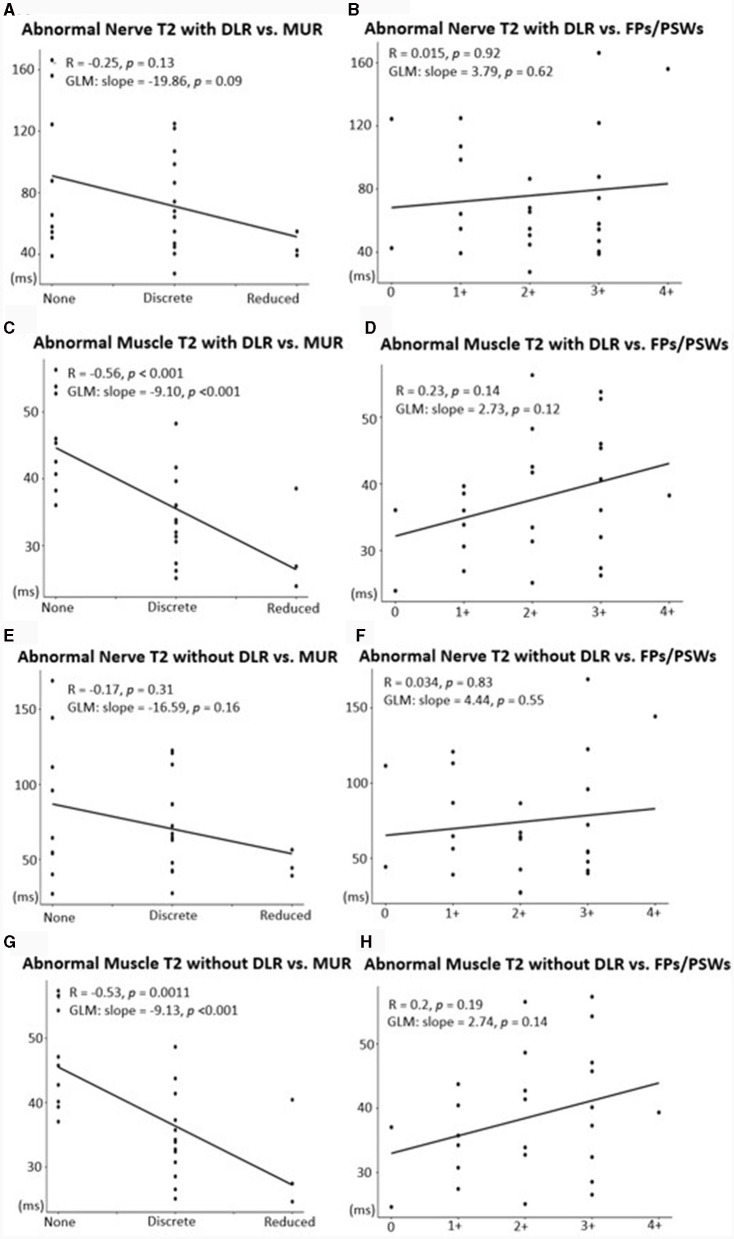
Associations between mean T2 with DLR **(A–D)** or with standard reconstruction **(E–H)** and EMG metrics of denervation potentials [fibrillation potentials (FPs)/positive sharp waves (PSWs)] and motor unit recruitment (MUR). A significant, positive association was shown between abnormal nerves and MUR **(A, E)** and abnormal muscles and MUR **(C, G)**. Associations between abnormal nerves and FPs/PSWs **(B, F)** and between abnormal muscles and FPs/PSWs **(D, H)** were not significant.

The generalized linear model showed a significant association between increased MUR severity and higher DESS-T2 in muscle, both with DLR and standard reconstruction (DLR: 9.10 msec per MUR grade, *p* < 0.001; standard reconstruction: 9.13 msec per MUR grade, *p* < 0.001). In nerve, a higher T2 per MUR grade was also observed (DLR: 19.86 msec/grade, standard reconstruction: 16.59 msec/grade), but the association was not significant (DLR: *p* = 0.09, standard reconstruction: *p* = 0.16) (Figure 6). No significant association was found between DESS-T2 and FPs/PSWs (DLR: *p* = 0.62 for nerve, *p* = 0.12 for muscle; standard reconstruction: *p* = 0.55 for nerve, *p* = 0.19 for muscle).

### Magic angle dependency

A weakly positive correlation was found between the angle of normal nerves and mean DESS-T2 (slope = 0.19 msec/°, *r* = 0.18, *p* = 0.39) (Supplementary Figure 2). This association was negligible for abnormal nerves (*p* = 0.81).

## Discussion

The 3D-DESS MRN sequence facilitates T2 mapping for quantitative nerve and muscle assessment within a single 3D acquisition with high in-plane and through-plane spatial resolution in the elbow and forearm regions. Spatial registration between the anatomical DESS images and DESS-T2 maps was not required for analysis, as both DESS images and DESS-T2 maps derive from the same acquisition. The application of DLR decreased mean T2 of both normal and abnormal muscles, which is consistent with reduced Rician bias. These observations were, however, not significant in nerves, which might be due to their smaller cross-sectional areas leading to higher T2 variation, as compared to muscles.

DESS-T2 values for abnormal muscles and nerves were higher than those for normal muscles and nerves with both DLR and with standard reconstruction. These preliminary results suggest that DESS-T2 mapping might be useful for quantitative evaluation in peripheral neuropathies and could complement qualitative MRN evaluation. The increased T2 in muscles and nerves was also associated with MUR severity from EMG, which suggests that DESS-T2 mapping might indicate the degree of neuropathy. These associations parallel those found with conventional MESE T2 mapping of muscles in brachial plexopathies (7). However, similar associations were not found between T2 and measures of active denervation, which may relate to the range in days from symptom onset to EMG, as the onset and severity of denervation potentials vary with time (31).

Though nerves at the elbow are approximately parallel to B_0_ in both prone and supine positions, a range of angles between B_0_ and the analyzed nerves were observed in this study, from 6.14° to 46.10° (mean = 24.28° ± 9.89°). Therefore, it was reasonable to investigate the magic angle effect in this work; a correlation between the angle from B_0_ of the normal nerve and T2 was observed. Though this correlation was not significant, it was comparable to that observed previously with two-echo T2-mapping (36, 37). This weak correlation could be due to between-subject variation of nerve T2, even for normal nerves. The correlation was negligible for abnormal nerves, which may be due to variation in T2 due to the type and severity of neuropathy. The magic angle's impact on T2 measurements used for analysis will require further investigation in future studies that more comprehensively evaluate nerve trajectories. Importantly, such studies could be enabled by the high-resolution, 3D sequences used in this work.

DESS-T2 was overall lower than that derived from conventional MESE T2, except for abnormal nerves, and the correlations between DESS and MESE were significant for abnormal muscles and normal nerves. This suggests that DESS-T2 values could be compared to conventional T2 evaluation of nerves and muscles. The reasons for the underestimation of DESS-T2 include the use of fat suppression in DESS as compared to no fat suppression in MESE in this work, and few (only two) echoes in DESS available for T2 calculation. Moreover, DESS was acquired at a higher spatial resolution than MESE, which might be an additional source of T2 variation. Furthermore, DESS-T2 has been shown to be less B_1_-sensitive than MESE (33, 38), which might also explain differences in the T2 values obtained.

3D DESS-T2 with DLR provides a feasible way for high quality T2 mapping in nerve imaging, but other promising approaches for accelerating MESE acquisition that utilize T2 decay have been recently proposed (39, 40). As the current DLR approach does not use relaxation models, future incorporation of T2 decay with DLR may further improve results of 3D DESS-T2 maps.

### Limitations

While the sample size in this work was not high (*n* = 25), the study was adequately powered to observe differences between normal and abnormal nerves and muscles. This work was also performed at a single institution and at a single field strength. Furthermore, this work tested the DLR implementation from one vendor, and hence the generalizability of our work may not extend to other DLR implementations. As this work did not include longitudinal analysis, associations between T2 and the number of days from symptom onset were not analyzed. Additionally, as this was a retrospective study, there was a wide interval from EMG to MRI (range = −12 to +95 days, mean = 18.2 ± 25.78 days); all MRI were performed following EMG, except in one subject. Future prospective studies could target shorter intervals between EMG and MRI to ensure similar disease status. Associations between T2 and EMG metrics may also be confounded by inter-observer variability between EMG operators. This work did not correlate MR results with muscle strength testing to further establish the clinical relevance of these findings, as muscle strength results are sometimes unavailable and are frequently subjective. Future work could include a prospective study that includes same-day EMG and quantitative muscle dynamometry comparisons (for muscle strength) to MRI.

### Conclusion

3D-DESS generated T2 maps are improved with DLR, and 3D DESS-T2 can be used to quantitatively determine differences in T2 values of nerves and muscles involved in upper extremity peripheral neuropathies.

## Data availability statement

The raw data supporting the conclusions of this article will be made available by the authors, without undue reservation.

## Ethics statement

The studies involving humans were approved by Hospital for Special Surgery Institutional Review Board. The studies were conducted in accordance with the local legislation and institutional requirements. The ethics committee/institutional review board waived the requirement of written informed consent for participation from the participants or the participants' legal guardians/next of kin because of the study's retrospective nature.

## Author contributions

GC: Writing – original draft, Writing – review & editing, Conceptualization, Data curation, Investigation, Methodology, Project administration, Validation, Visualization. DS: Writing – original draft, Writing – review & editing, Conceptualization, Data curation, Funding acquisition, Investigation, Methodology, Supervision, Validation. SQ: Writing – original draft, Writing – review & editing, Data curation, Investigation, Project administration. YL: Writing – original draft, Writing – review & editing, Data curation, Investigation, Supervision. QL: Writing – original draft, Writing – review & editing, Formal analysis. ET: Writing – original draft, Writing – review & editing, Conceptualization, Data curation, Formal analysis, Funding acquisition, Investigation, Methodology, Project administration, Resources, Software, Supervision, Validation, Visualization.
