# Supplementary material for: Quantitative double echo steady state T2 mapping of upper extremity peripheral nerves and muscles
Source: Front Neurol. 2024 Feb 15;15:1359033. doi: 10.3389/fneur.2024.1359033 (PMC10902120; doi:10.3389/fneur.2024.1359033)
Supplement: Supplementary file 1 [file Table_1.pdf]

Supplementary Table 1. MRI parameters, including a qualitative 2D MRN sequence not analyzed in this work.

**Sequence**	**3D DESS**	**2D MESE (Conventional T2 Mapping)**	**2D T_2w-FSE**
Qualitative/Quantitative	Both	Quantitative	Qualitative
Scan time (min:sec)	4:00-6:00	5:00	4:12
Orientation	Axial	Axial	Axial
Field-of-view (cm)	9-12	14-16	12-14
TR/TE (msec)	15/5-10	2200/11-88 (8 echoes)	3500/85
Matrix size (frequency x phase)	320 x 320	256 x 224	320 x 224
Slice thickness (mm), Spacing	1.6, -	4.0,1.0	2.5-3.5, -
# Slices	72-90	22	42-64
Bandwidth (kHz)	±41.67	±15.63	±31.25
Echo train length	2	8	14
Flip Angle (^{o})	35	-	-
Fat Suppression	Spectral spatial	None	Dixon

Double echo steady state (DESS), multi-echo spin echo (MESE), fast spin echo (FSE), magnetic resonance neurography (MRN)
