# Supplementary material for: Quantitative double echo steady state T2 mapping of upper extremity peripheral nerves and muscles
Source: Front Neurol. 2024 Feb 15;15:1359033. doi: 10.3389/fneur.2024.1359033 (PMC10902120; doi:10.3389/fneur.2024.1359033)
Supplement: Supplementary file 2 [file Image_1.pdf]

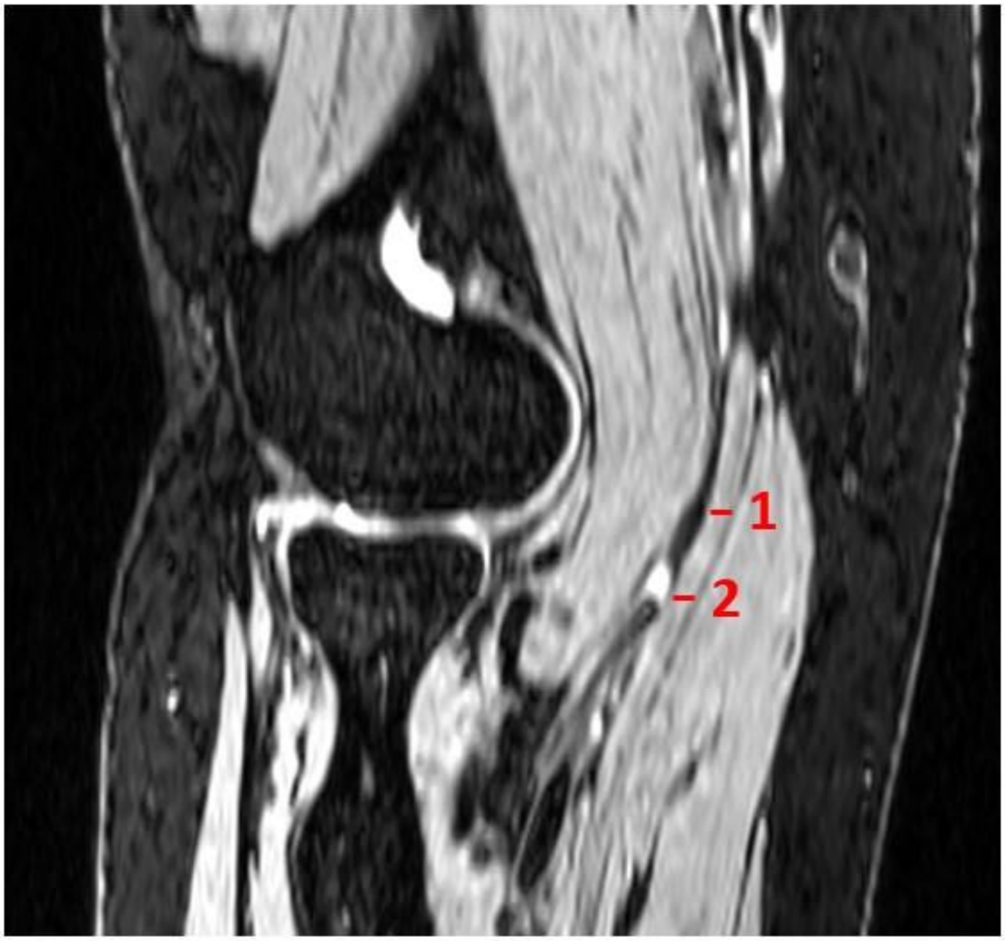

$$(x_1, y_1, z_1)$$
$$(x_2, y_2, z_2)$$
$$\text{Angle} = \cos^{-1} \frac{z_2 - z_1}{\sqrt{(z_2-z_1)^2+(x_2-x_1)^2+(y_2-y_1)^2}}$$

Supplementary Figure 1. Oblique coronal DESS S- image from a 47-year-old woman with Parsonage-Turner syndrome shows the selection of coordinates at two locations (1 and 2) along the course of the ulnar nerve at the elbow for calculating the angle of the nerve relative to B_0. Vector coordinates ((x_1, y_1, z_1) and (x_2, y_2, z_2)) were used to calculate the angle of the nerve with respect to B_0 according to the equation below.
