# Supplementary material for: Quantitative double echo steady state T2 mapping of upper extremity peripheral nerves and muscles
Source: Front Neurol. 2024 Feb 15;15:1359033. doi: 10.3389/fneur.2024.1359033 (PMC10902120; doi:10.3389/fneur.2024.1359033)
Supplement: Supplementary file 3 [file Image_2.pdf]

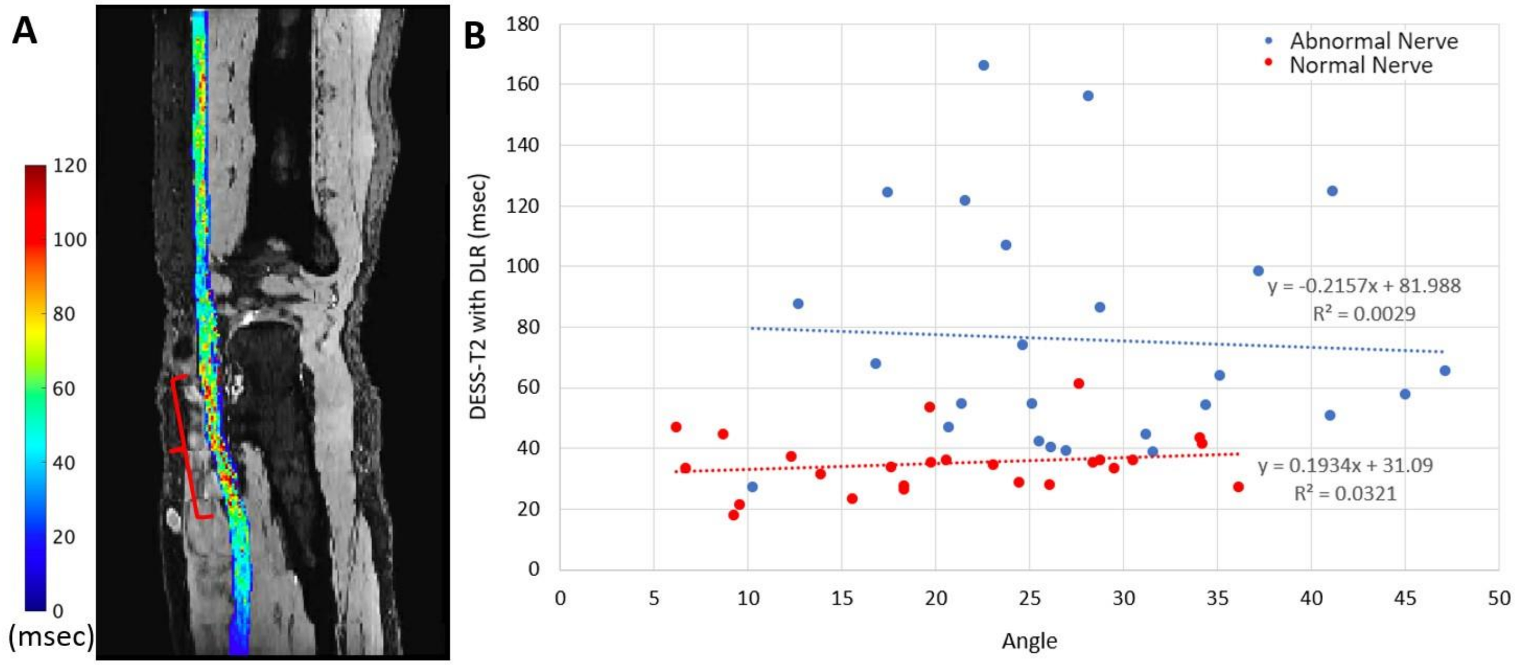

Supplementary Figure 2. 3D DESS oblique coronal multiplanar reformatted image, with T2 map overlay **(A)**, from a 67-year-old male subject with ulnar neuropathy demonstrates elevated T2 of the ulnar nerve as it changes its angle through the cubital tunnel (arrow). Linear regression analysis of the angle relative to B_0 vs. T2 values in all patients (N=25) suggests a positive but weak correlation for the normal nerve and a negligible correlation for abnormal nerve **(B)**.
